# Spine Injuries in Household Environments: A Comprehensive Analysis

**DOI:** 10.7759/cureus.44275

**Published:** 2023-08-28

**Authors:** Gurbinder Singh, Varun Rao, Aish Thamba, Bhavya Pahwa, Mohamed Zaazoue

**Affiliations:** 1 Department of Orthopaedic Surgery, University of California-San Francisco School of Medicine, San Francisco, USA; 2 Department of Neurological Surgery, Indiana University School of Medicine, Indianapolis, USA; 3 Medical School, University College of Medical Sciences, New Delhi, Delhi, IND

**Keywords:** trauma and injury prevention, age-specific risks, gender-based differences, home locations, spine injuries

## Abstract

Introduction

Recognizing the concerns posed by spine injuries within homes, stemming from falls, interactions with furnishings, and daily activities, it is imperative to consider preventive strategies. Our analysis of spine injuries utilizing the National Electronic Injury Surveillance System (NEISS) data sheds light on falls, furnishings, age-specific risks, recreation, technology, and socioeconomic disparities as contributing elements, accentuating the need for targeted interventions. This study aims to provide insights into the prevalence of spine injuries in different household locations, associated products, age groups, and gender, thus informing injury prevention strategies for safer living environments.

Methods

This is a retrospective, cross-sectional study utilizing data between 2013 to 2022 from the National Electronic Injury Surveillance System database. Specific household product codes and demographic data, such as age and gender, were analyzed. Statistical analysis in R (R Foundation for Statistical Computing, Vienna, Austria) involved descriptive statistics and multivariate logistic regressions.

Results

In analyzing 44,267 spine injuries, the study revealed location-specific variations in spine injuries within households. Living rooms and bedrooms had the highest injury rates at 34.17% and 21.65%, respectively. Significant differences in injury rates between males and females across various home locations. Females accounted for 51.78% of injuries in the living room and 59.99% in the bedroom. In the kitchen, females experienced 53.21% of injuries, while males accounted for 46.79% of cases. Notably, overall spine injuries exhibited a significant difference between males and females, with females having a higher total likelihood of injuries (AOR = 1.21, 95% CI: 1.14-1.77, p < 0.001). Regarding age, individuals between 51-60 years were most vulnerable to spine injuries, accounting for 17.98% of total cases. Notably, the age group of 61-70 years exhibited a substantial proportion of injuries at 17.12%, while the age group of 71-80 years accounted for 14.39%. The age group of 41-50 years also displayed a notable injury rate of 14.12%. The youngest age group, 0-10 years, demonstrated the lowest percentage of injuries at 4.79%. This age-based analysis provides valuable insights into the distribution of spine injuries across different demographic segments.

Regarding age, individuals between 51-60 years were most vulnerable to spine injuries, comprising 17.98% of total cases. Age groups of 41-50 and 61-70 years also showed substantial proportions of injuries, accounting for 14.12% and 17.12%, respectively. The youngest age group, 0-10, exhibited the lowest percentage of injuries at 4.79%.

Conclusion

The study focuses on the occurrence of spinal injuries in common sites of injury in the household, such as the living room, bedroom, kitchen, and stairs. There is increased prevalence amongst females and increased risk vulnerability amongst people 51 to 60 years of age. Our research emphasizes the necessity of implementing specific injury prevention measures tailored to different demographic groups within their home setting. This approach should involve collaborative decision-making with patients while prioritizing patient education to create a safer living environment and reduce the likelihood of spine injuries.

## Introduction

Spinal injuries that occur within the confines of one's own home pose a serious threat to public health and lead to considerable levels of injury and healthcare expenses, with costs per patient exceeding one million US dollars [1]. A highly useful resource in studying non-life-threatening injuries treated within American hospital emergency departments is the National Electronic Injury Surveillance System (NEISS), operated by the U.S. Consumer Product Safety Commission [2]. Researchers have shown an increasing interest in comprehending the epidemiology surrounding spinal injuries that occur at home as a means of guiding prevention strategies and implementing effective interventions [3-8].

Numerous inquiries have shed light on the importance of comprehending the underlying factors that contribute to these injuries. For instance, a certain study discovered that slipping, tripping, stumbling, and tumbling down from structures like buildings, stairs, and steps are the primary culprits behind spinal injuries caused by falls in residential areas. Furthermore, this examination showed that individuals aged 61 years or more are particularly prone to experiencing falls on the same level, which subsequently result in severe functional spinal injuries at the cervical and thoracic levels. This underscores the imperative need for targeted fall prevention approaches specifically designed for the elderly population [3,6,9]. 

Items in the home environment are significant factors contributing to spine injuries [6,10]. Items such as step stools and ladders were found to increase the risk of falls resulting in spinal trauma. This highlights the urgency for safety education and adjustments in product design [4,6,8,10-13]. To better understand susceptibility to spine injuries within different age groups at home, researchers have conducted studies and discovered that elderly individuals residing independently face hazards like loose carpets and insufficient lighting. Thus, it is important to conduct thorough assessments of home safety and implement necessary modifications tailored specifically for older adults [8,10,14,15]. 

The rise of recreational activities and sports in the household environment has also created unique events leading to spine injuries [7]. Trampoline use has accounted for a substantial proportion of spine injuries in children and adolescents, emphasizing the need for safety guidelines and adult supervision while using trampolines [16]. Similarly, the widespread adoption of electronic devices has increased spine injuries related to their use at home. A condition known as text neck syndrome, resulting from excessive forward head flexion while using mobile devices, has seen a concerning increase, particularly among adolescents and young adults [17].

In addition, the social determinants of health play a role in the burden of home-related spine injuries. Individuals from lower-income households experience higher rates of such injuries, which emphasizes the need for injury prevention strategies tailored specifically for these populations [7,18,19]. We hypothesize that spine injuries within residential settings exhibit different patterns depending on where individuals are affected, how they were injured, and their age groups. Our expectation is to observe variations and identifiable trends in the distribution of types of spine injuries, mechanisms of injury, and affected age groups. This will provide valuable insights into the complex nature of spinal injuries occurring in domestic environments.

Spine injuries that occur in homes are a matter of great concern when it comes to public health. Our study, which relies on the NEISS database, presents a thorough examination shedding light on the epidemiology, risk factors, and preventive measures connected to these injuries. Having knowledge about these contributing factors is crucial for creating interventions with specific goals in mind, encouraging safety practices, and ultimately reducing the occurrence of spine injuries within residential environments.

## Materials and methods

A cross-sectional design examined total spine injuries occurring within households across different age groups over ten years from 2013 to 2022. The primary data source for this investigation was the National Electronic Injury Surveillance System (NEISS) database, which has been previously utilized in several published studies [20,21]. This dataset provides valuable, de-identified, nationally representative, and demographically diverse information on injuries treated within emergency departments of approximately 100 hospitals across the United States carefully selected as a probability sample of all 6100 United States hospitals with a minimum of six beds and a 24-hour emergency department. The data is anonymized, cannot be linked to specific individuals, and is exempted from Institutional Review Board approval by falling into the exempt human subject research category. 

Our study's initiation involved an initial count of 48,263 entries, specifically focused on spine injuries. We systematically excluded entries that were missing an associated product code. To ensure a contemporary analysis, we focused on data from the previous decade. Within this timeframe, we gathered spine injury data from each year, culminating in an analysis sample of N=44,267. Our database search encompassed diagnoses related to spine injuries categorized under the body part code "spine" from 2013 to 2022. Our research team then meticulously selected relevant product codes linked to various household environments, including "kitchen," "living room," "bedroom," "stairs," "outdoor," and "other," for further investigation.

In the “kitchen” category, items such as toasters, ice crushers, ice cream makers, coffee makers, electric blenders, electric frying pans, electric can openers, electric waffle irons, bread-making machines, and kitchen gadgets were included in the analysis. The “living room” category included items such as sofas, couches, tables, entertainment centers, TV stands, recliners, armchairs, media consoles, coffee tables, end tables, picture frames, mirrors, floor lamps, table lamps, wall art, magazine racks, and decorative items. The “bedroom” category included items such as mattresses, bedding, bedspreads, throws, bunk beds, toddler beds, bed rails, bedside tables, dressers, wardrobes, full-length mirrors, jewelry boxes, alarm clocks, pillows, and decorative cushions. The “stairs” category included items included stair gates, staircases, stair treads, balusters, newel posts, stair risers, stair stringers, stair nosing, stair handrail brackets, stair carpet pads, stair mats, stair carpet or runners, stair slide or play accessories, stair gates for child safety, and stair railing kits. The “outdoor” category included items such as garden tools, barbecue grills, garden tractors, snow blowers, patio furniture, outdoor kitchen equipment, treehouses, garden furniture, outdoor umbrellas, outdoor heaters, garden sprayers, outdoor lighting, outdoor storage, outdoor decor, outdoor water features, outdoor pet houses, outdoor power tools, outdoor sports equipment, outdoor safety equipment, outdoor insect traps, and outdoor surveillance cameras. The “other” category included items such as decorative items, clocks, curtains, blinds, shades, curtains, indoor safety equipment, indoor plants, indoor exercise equipment, indoor games, indoor decorative lighting, indoor storage solutions, indoor sports equipment, indoor entertainment systems, indoor water features, indoor games, and various home-related products such as first aid kits, emergency kits, home office furniture and equipment, laundry appliances, and home automation devices. This approach allowed diverse analysis to occur across different living spaces, providing valuable insights into spine injuries localized to specific environments in the home. 

For each spine injury, the age group and gender of the individual were extracted and analyzed to provide demographic associations in the prevalence of spine injuries per prior literature [20-23]. The data was cleaned with R programming language 4.3.1 (R Foundation for Statistical Computing, Vienna, Austria) to ensure accuracy and consistency. Duplicate entries and incomplete records were meticulously reviewed and removed to ensure the accuracy and reliability of the findings. The data was then organized and stratified based on household locations to permit the analysis of the distribution of spine injuries across different areas of the home.

Descriptive statistics were employed to analyze and present the patterns and trends associated with spine injuries and home location, sex, and age group. Similar to prior studies, sex patterns were examined using multivariate logistic regression and were adjusted for age in the analysis [20,24-27]. Adjusted odds ratios (AOR) with their corresponding 95% confidence intervals (CI) and p-values were calculated to determine the significance of the association. Values of AOR greater than one signified increased odds of the spine injury being linked to the female sex, relative to the reference group (males). AOR values less than 1 indicate decreased odds of a female sustaining a spine injury compared to males.

## Results

Table 1 presents the descriptive statistics for the analytic sample (N = 44,267), with the living room being the most common site of injuries at 34.17% of total cases. The bedroom followed with 21.65% of injuries, while the kitchen and stairs accounted for 12.52% and 16.70%, respectively. Outdoor areas and other spaces had lower injury rates at 9.47% and 5.53% of cases, respectively. 

**Table 1:** Home Locations and Frequency of Spine Injuries The table displays the number of spine injuries and the percentage of total injuries for each home location.

Home Location	Number of Injuries	% of Total Injuries
Living Room	15128	34.17%
Bedroom	9584	21.65%
Stairs	7392	16.70%
Kitchen	5544	12.52%
Outdoor (Yard/Patio)	4169	9.42%
Other	2450	5.53%
Total Spine Injuries	44267	100%

Table 2 explores the distribution of spine injuries by sex and home locations, revealing significant differences between male and female injury rates across different areas. In the living room, females had slightly higher injury rates than males, accounting for 51.78% of the injuries, whereas males comprised 48.22% of the cases. Similarly, in the bedroom, females accounted for 59.99% of the injuries, and males made up 40.01% of the cases. Females demonstrated higher injury rates in both the kitchen and outdoor areas, comprising 53.21% and 61.14% of the cases, respectively, while males accounted for 46.79% and 38.86% of the injuries in these specific locations. The total number of spine injuries for males was 19,039, representing 44.3% of all cases, while females had 23,939 injuries, making up 55.70% of the total. 

**Table 2:** Distribution of Spine Injuries by Sex and Home Locations The table presents the number of spine injuries in each home location for male and female individuals.

Variable	Male (ref)	Female	AOR (95% CI)	P-value
Living Room	7301 (48.22%)	7838 (51.78%)	1.11 (.92-1.83)	0.192
Bedroom	3836 (40.01%)	5748 (59.99%)	1.54 (1.22-1.61)	<0.001
Kitchen	2594 (46.79%)	2950 (53.21%)	1.07 (0.95-1.13)	0.364
Stairs	2893 (39.11%)	4499 (60.89%)	1.47 (1.06-1.82)	<0.001
Outdoor (Yard/Patio)	1619 (38.86%)	2549 (61.14%)	1.13 (.94-1.24)	0.213
Other	798 (32.57%)	1652 (67.43%)	1.29 (1.11-1.53)	<0.001
Total Spine Injuries	19039 (44.3%)	23939 (55.70%)	1.21 (1.14-1.77)	<0.001

In the living room, females showcased a slightly higher probability of spine injuries than males (AOR = 1.11, 95% CI: 0.92-1.83, p = 0.192), though this outcome did not achieve statistical significance. However, notable distinctions emerged in the bedroom (AOR = 1.54, 95% CI: 1.22-1.61, p < 0.001), stairs (AOR = 1.47, 95% CI: 1.06-1.82, p < 0.001), and other spaces (AOR = 1.29, 95% CI: 1.11-1.53, p < 0.001), where females exhibited increased likelihoods of injuries in comparison to males. No statistically significant differences were observed in the kitchen and outdoor(yard/patio) areas. Spine injuries across all locations demonstrated a noteworthy difference between males and females (AOR = 1.21, 95% CI: 1.14-1.77, p < 0.001), underscoring the increased probability of spine injuries among females.

Table 3 provides a comprehensive overview of the relationship between home locations and age groups concerning the occurrence of spine injuries. The age group of 51-60 years exhibited the highest injury percentage, constituting 17.98% of the total cases. Additionally, age groups of 61-70 years and 71-80 years displayed substantial proportions of injuries, accounting for 17.12% and 14.39%, respectively. In contrast, the age group of 41-50 years presented a lower percentage of injuries at 14.12%. The youngest age group, 0-10 years, exhibited the lowest percentage of injuries at 4.79%. The remaining age groups of 11-20, 21-30, and 81 years and above showcased varying levels of injury rates.

**Table 3:** Home Locations of Spine Injuries and Age The table illustrates the percentage of spine injuries for different age groups in each home location.

Home Location	0-10 years	11-20 years	21-30 years	31-40 years	41-50 years	51-60 years	61-70 years	71-80 years	81 years and above
Living Room	5.53	7.57	7.42	12.02	13.32	20.71	16.39	12.39	4.65
Bedroom	6.36	4.21	12.59	12.60	15.41	18.77	14.48	10.61	4.97
Kitchen	4.12	6.24	5.16	11.24	15.93	18.04	16.69	14.72	7.86
Stairs	5.20	7.04	8.67	11.81	14.89	17.80	14.65	15.47	4.47
Outdoor (Yard/Patio)	4.37	3.03	7.35	10.33	13.88	17.38	18.56	16.52	8.58
Other	3.15	5.28	3.67	8.76	11.29	15.18	17.96	16.62	18.09
Total Spine Injuries	4.79	7.06	9.68	11.13	14.12	17.98	17.12	14.39	3.73

**Figure 1:**
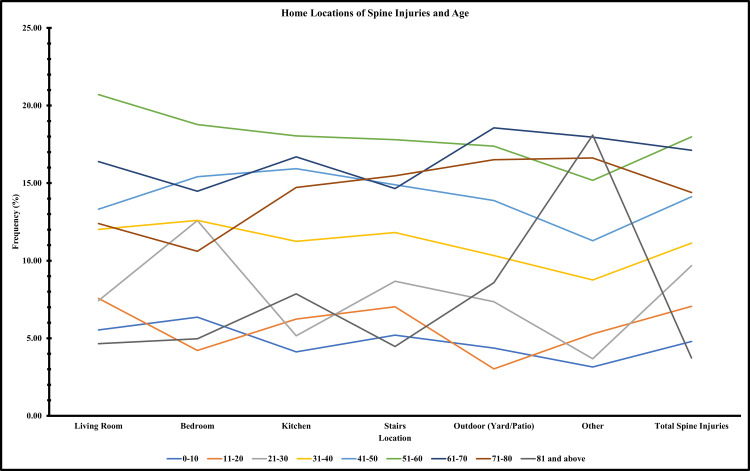
Spine Injuries by Home Locations and Age Groups This figure illustrates the percentage of total injuries in different home locations across various age groups.

To visually represent these findings, the graph in Figure 1 demonstrates injury patterns across various home locations for different age groups. This graph highlights fluctuations in injury occurrence within each site for different age segments.

## Discussion

This study provides a comprehensive investigation into spinal injuries that occur in residential settings, with a particular emphasis on various factors including location, gender, and age groups. By delving further into these aspects of analysis, it is uncovered that living rooms witness the highest occurrence of such injuries, making up 34.17% of the total reported cases (Table 1). In comparison, bedrooms rank second in terms of injury frequency, accounting for 21.65% of all documented incidents (Table 1).

Furthermore, it becomes evident that females constitute a significant proportion of the documented cases while varying degrees of injury rates are observed across different areas within households based on gender-specific patterns. Furthermore, age emerges as a crucial determining factor; individuals between the ages of 51-60 years experience the highest percentage of injuries irrespective of their location within homes (Table 3). Conversely, the youngest age group (0-10 years) exhibits the lowest rate due to increased supervision and safety measures being put in place.

Figure 1 provides an illustration of the trends in injury occurrences within different areas of homes for individuals of varying age groups. This suggests the importance of implementing tailored measures that consider both the specific location and age group at risk. The data shows that the living room is the most frequent site for these injuries, closely followed by the bedroom, kitchen, and stairs areas. This implies that activities and potential dangers in these spaces play a significant role in causing spinal injuries. On the contrary, outdoor spaces and other unspecified locations demonstrate lower rates of injuries related to spinal issues, indicating a relatively lesser risk compared to other parts of preventive measures against spine injuries. 

Furthermore, when examining gender-specific patterns, it is evident that females report more instances of spine injuries than males. There are variations regarding where these injuries occur based on gender, suggesting potential risks tied to specific sections of households designed for each gender. For instance, females experience significantly higher odds of injury in other undesignated areas outside, bedrooms, and stairs when compared to males (Table 2). This could be partially explained by post-menopausal osteoporosis since the osteoporosis disease process has been implicated in a higher rate of traumatic spinal vertebral injuries through the mechanism of fragility fractures. Recognizing these disparities between genders can aid in customizing injury prevention strategies to meet distinct needs, such as earlier screening measures and subsequent treatment of osteoporosis [28].

The prevalence of injuries reveals that age plays a pivotal role, with the highest occurrence among individuals aged 51-60. This highlights the importance of addressing age-related vulnerability when creating preventative strategies. Although younger age groups, particularly children aged 0-10, experience fewer injuries, this can be attributed to increased vigilance and safety measures implemented for them at home. However, the diverse injury rates across different age brackets provide valuable insights for designing interventions and safety guidelines specific to each age group. Additionally, it is crucial to consider age-specific susceptibility when implementing preventive measures. Older individuals within the 51-70 range appear to face higher risk and thus necessitate interventions tailored precisely according to their unique safety needs [3,5,29]. Nevertheless, the diverse injury rates across other age groups offer insights for creating interventions and safety guidelines that cater to specific age ranges [4,9,12,19].

Additionally, adopting a multidisciplinary approach becomes imperative to risk stratify accurately as well as assess and manage adults living within community settings. Improving environments free of poor lighting, lack of a grab rail, and unsafe climbing on chairs and ladders while addressing underlying functional impairment reduces the risk of falls in older adults. Patient education, physiotherapy referral, and encouraging patient involvement in addressing risk factors, capacity, and behaviors for falls have been effective interventions, as well [4-6,8,11,14,29,30]. 

With regards to the youngest age group, encompassing individuals aged 0 to 10 years old, even though the rates of injury are lower, it is imperative that efforts in prevention be focused on creating a safe environment and enhancing supervision. This will help in decreasing the likelihood of spine injuries. 

Recognizing the limitations of this study is vital, including its dependence on information obtained from hospital emergency departments. These departments may not accurately document all cases of spinal injuries occurring in homes, potentially skewing the results. Moreover, it's important to note that the NEISS database lacks comprehensive data on the specific mechanisms and circumstances surrounding these injuries. This missing information hinders a thorough comprehension of the pathophysiology underlying spinal injury occurrences.

This research presents valuable insights into the frequency of spinal injuries in residential settings. Additionally, it introduces options for adopting evidence-based approaches like motivational interviewing, home repairs, patient education, and shared decision-making to establish safer living spaces and ultimately reduce the chances of spinal injuries. To further alleviate the prevalence and impact of spinal injuries at home, future research should explore other risk factors and potential interventions [6,10].

## Conclusions

This study provides valuable insights into spine injuries in households, focusing on home locations, sex, and age groups. The living room, bedroom, kitchen, and stairs are common injury sites, warranting targeted preventive measures. Females experience more cases, and injury rates vary among home locations. Age-specific vulnerability is evident, with the 51-60 age group at highest risk. Tailored interventions for specific areas and demographics are crucial for a safer living environment. Our findings suggest that evidence-based strategies such as motivational interviewing, patient education, home repair, and shared decision-making would reduce spine injuries in household environments.
